# Ingestion and sublethal effects of physically and chemically dispersed crude oil on marine planktonic copepods

**DOI:** 10.1007/s10646-014-1242-6

**Published:** 2014-04-23

**Authors:** Rodrigo Almeda, Sarah Baca, Cammie Hyatt, Edward J. Buskey

**Affiliations:** 1 Marine Science Institute, University of Texas at Austin, 750 Channel View Drive, Port Aransas, TX 78373 USA; 2 College of Sciences, University of Texas at El Paso, El Paso, TX USA

**Keywords:** Crude oil, Dispersant Corexit 9500A, Planktonic copepods, Sublethal toxic effects, DWH crude oil spill, Environmental pollution

## Abstract

Planktonic copepods play a key function in marine ecosystems, however, little is known about the effects of dispersants and chemically dispersed crude oil on these important planktonic organisms. We examined the potential for the copepods *Acartia tonsa*, *Temora turbinata* and *Parvocalanus crassirostris* to ingest crude oil droplets and determined the acute toxicity of the dispersant Corexit^®^ 9500A, and physically and chemically dispersed crude oil to these copepods. We detected ingestion of crude oil droplets by adults and nauplii of the three copepod species. Exposure to crude oil alone (1 µL L^−1^, 48 h) caused a reduction of egg production rates (EPRs) by 26–39 %, fecal pellet production rates (PPRs) by 11–27 %, and egg hatching (EH) by 1–38 % compared to the controls, depending on the species. Dispersant alone (0.05 µL L^−1^, 48 h) produced a reduction in EPR, PPR and EH by 20–35, 12–23 and 2–11 %, respectively. Dispersant-treated crude oil was the most toxic treatment, ~1.6 times more toxic than crude oil alone, causing a reduction in EPR, PPR and EH by 45–54, 28–41 and 11–31 %, respectively. Our results indicate that low concentrations of dispersant Corexit 9500A and chemically dispersed crude oil are toxic to marine zooplankton, and that the ingestion of crude oil droplets by copepods may be an important route by which crude oil pollution can enter marine food webs.

## Introduction

Petroleum or crude oil pollution in the sea is a growing, major environmental problem. During the last decades, the rise in world energy demand and the growing use of petroleum products have resulted in intensified exploration, production and transportation of petroleum in the sea, making marine environments especially susceptible to increased risk of crude oil spills (National Research Council, NRC 2003; Dalsøren et al. 2007). Large accidental crude oil spills are not the most important source of petroleum discharge into the marine environment (NRC 2003), but the sudden discharge of high concentrations of crude oil in the sea has strong short- and long-term harmful environmental impacts (Kennish 1996). The recent Deepwater Horizon (DWH) Oil Spill in the Gulf of Mexico (2010), the world’s largest accidental release of crude oil into the ocean in history (National Commission on the BP Deep Ocean Horizon Oil Spill and Offshore Drilling 2011), is a clear example of the dramatic ecological and economic consequences of marine crude oil spills (Barron 2012; Sumaila et al. 2012; White et al. 2012). Among the biological components of marine ecosystems, planktonic communities are particularly susceptible to crude oil pollution (Walsh 1978; Graham et al. 2010; Ortmann et al. 2012). Among marine plankton organisms, copepods are the dominant components of mesozooplankton and probably the most abundant metazoans on Earth (Longhurst 1985; Humes 1994). Planktonic copepods play a crucial role in the transfer of matter from low to higher trophic levels in marine food webs (Banse 1995; Verity and Smetacek 1996) and they are the main prey of many species of fish and fish larvae (Last 1980), contributing decisively to the recruitment of fish stocks of commercially important species (Castonguay et al. 2008). However, despite the ecological importance of copepods, our knowledge of the effects of dispersed crude oil on planktonic copepods is still very limited. Many crude oil toxicology studies on copepods have been focused on lethal effects (Jiang et al. 2010, 2012) whereas the sublethal effects have been less frequently investigated. Sublethal effects of crude oil such as reduced copepod egg production and hatching may have important implications for secondary production in the pelagic environment. Therefore, determining sublethal effects of crude oil on marine copepods is necessary to accurately evaluate the effects of oil spills on planktonic communities.

During the DWH oil spill, more than 7 million liters of chemical dispersants, mainly Corexit^®^ 9500A, were released in the Gulf of Mexico to treat the crude oil spill (TFISG-OBCSET, The Federal Interagency Solutions Group, Oil Budget Calculator Science and Engineering Team 2010). More than 4 million liters were applied to the sea surface and ca. 3 million liters to the subsea at the DWH well head (TFISG-OBCSET 2010). This is the largest known application of chemical dispersants in the sea in response to a crude oil spill (Wise and Wise 2011). Dispersants are commonly used for crude oil spill clean-up because they promote the formation of small crude oil droplets (Canevari 1978; Clayton et al. 1993), enhancing their rate of natural dispersion, and reducing the risk of oil slicks arriving to coastal areas and physical contamination (smothering; US Environmental Protection Agency, EPA 2010). The dispersants used during DWH oil spill, mainly Corexit^®^ 9500A, are less toxic than the older types of dispersants, which were extremely toxic and caused drastic negative impacts on marine life as observed in the aftermath of the Torrey Canyon (1967) and Sea Empress (1966) crude oil spills (Corner et al. 1968; Nelson-Smith 1968; Swedmark et al. 1973). However, the toxicity and environmental impact of Corexit 9500A are not fully known (Wise and Wise 2011). For instance, little is known about the toxic effects of Corexit 9500A dispersant and Corexit 9500A chemically dispersed crude oil on planktonic copepods despite their key function of these organisms in marine ecosystems. Recent evidence suggests that mesozooplankton communities from the Gulf of Mexico are strongly affected by Corexit 9500A treated crude oil (Almeda et al. 2013a). These results emphasize the need for more detailed studies on the effects of this type of dispersant on planktonic copepods to better evaluate the ecological consequences of using chemical dispersant for cleaning crude oil spills.

After a crude oil spill, small crude oil droplets (1–100 µm) generated by wind and waves and/or by treatment with chemical dispersants are effectively suspended in the water column (Canevari 1978; Lichtenthaler and Daling 1985; Delvigne and Sweeney 1988; Mukherjee and Wrenn 2009). These crude oil droplets are frequently in the food size spectra of many zooplankters, including planktonic copepods and there is evidence of ingestion of crude oil droplets by some zooplankton species (Conover 1971; Mackie et al. 1978; Hebert and Poulet 1980; Lee et al. 2012). However, the importance of the ingestion of dispersed crude oil by zooplankton has been traditionally ignored and considered as an anecdotic event. In fact, most research on crude oil toxicity on copepods has been conducted using the crude oil water soluble fraction (Barata et al. 2005; Calbet et al. 2007; Saiz et al. 2009; Jiang et al. 2010, 2012) without considering the ingestion of crude oil droplets as a potential mechanism/route affecting the toxicity of petroleum hydrocarbons to marine zooplankton.

In this study we examined the potential of marine planktonic copepods to ingest crude oil droplets and estimated the acute toxicity of physically and chemically dispersed Louisiana light sweet crude oil and the dispersant Corexit 9500 to these zooplankters. For this purpose, we determined the effects of exposure to crude oil alone, dispersant alone, and dispersant**-**treated crude oil on the survival, egg production rates (EPRs), egestion rates and egg hatching (EH) of three calanoid copepods, *Acartia tonsa*, *Temora turbinata* and *Parvocalanus crassirostris*. We hypothesized that: (1) the studied species of copepods and nauplii ingest crude oil droplets because crude oil droplets are likely to be in their prey size spectra and (2) copepods will experience substantial sublethal effects at low concentrations of dispersant and dispersed crude oil due to the toxicity of the chemical dispersant. The copepod species studied here belong to some of the most representative genera of coastal planktonic copepods and have important differences in spatial and temporal distribution (Razouls et al. 2005–2013). *A. tonsa* is a widespread species with an almost worldwide distribution in estuarine and coastal subtropical and temperate waters including the east coast of the USA and Gulf of Mexico, where they frequently are the dominant copepod species most of the year (Heinle 1966). *T. turbinata* is widely distributed from tropical to temperate waters of the Atlantic, Pacific, and Indian Oceans, and may be the dominant mesozooplankton species seasonally in tropical, coastal, and oceanic waters of the Gulf of Mexico and Caribbean Sea (López-Salgado and Suárez-Morales 1998). *P. crassirostris* is a small calanoid copepod widely distributed mainly in tropical and subtropical shelf and coastal waters, including the Gulf of Mexico (Johnson and Allen 2005).

## Materials and methods

### Experimental organisms

Zooplankton samples were collected from the Aransas Ship Channel near the University of Texas Marine Science Institute (MSI) or from a nearby channel in Corpus Christi Bay (Port Aransas, Texas) using a plankton net (150 µm mesh, 50 cm diameter) in 2013. Plankton samples from the Corpus Christi Bay Channel were collected by towing the plankton net through the surface water, whereas samples from the Aransas Ship Channel were collected from surface waters by tying a plankton net to the MSI pier and allowing it to stream with the tidal current for approximately 5–10 min. Specimens of *A. tonsa* were isolated from samples collected in the Corpus Christi Bay Channel in July and in the Aransas Ship Channel in October, when the tidal current was ebbing from the bays to the Gulf of Mexico. *T. turbinata and P. crassirostris* were isolated from zooplankton samples taken in July and October from the Aransas Ship Channel on flood tides from the Gulf of Mexico. The contents of the collection buckets (cod ends) were diluted into a plastic container containing whole seawater and kept in a cooler until returning to the laboratory. Once in the laboratory, the plankton samples were then screened through a 2,000 µm mesh sieve to remove large zooplankton and were kept in fresh seawater with aeration. Then, aliquots of the samples were examined under a dissecting microscope and adults of each species of copepods were identified and gently sorted from their respective plankton samples using a borosilicate glass pipette. Adults (males and females) of each species were held in groups (20–50 specimens, depending on the experiments) in small plastic beakers or petri dishes with 0.2 µm-filtered sea water (FSW) until the experiment began (<2 h). During the experiments copepods were fed with a mixture a cultured phytoplankton species (Table 1). Phytoplankton cultures were grown in f/2 culture medium prepared with 0.2 μm filtered sterilized natural seawater collected from Aransas Ship Channel. Phytoplankton cultures were held in 250 mL polycarbonate flasks at 20 °C and 34–35 ppt salinity on a 12:12 h light:dark cycle with cool-white fluorescent lights at an irradiance of approximately 25 μmol photons m^−2 ^s^−1^.

**Table 1:** Experimental conditions in the exposure experiments conducted with different species of planktonic copepods (*Acartia tonsa*, *Temora turbinata*, *Parvocalanus crassirostris*), including concentration of copepods (Conc.), temperature (*T*), phytoplankton species used as food (prey), prey concentration (prey conc.) and total prey carbon biomass (prey biomass)

Species/experiment	Conc. (Ind. L^−1^)	Sex ratio (female:male)	*T*	Prey	Prey conc. (cells mL^−1^)	Prey conc. (µg C mL^−1^)
*A. tonsa_*July	40	1.2:1	25.1	*Rhodomonas* sp. *Heterocapsa* sp.	30,000 1,500	1.3
*A. tonsa_*Oct	40	3:1	24.5	*Rhodomonas* sp. *Heterocapsa* sp.	30,000 2,500	1.6
*T. turbinata*	20	1:1	24.3	*Rhodomonas* sp. *T. weissflogii* *Heterocapsa* sp.	5,000 2,500 2,500	1.2
*P. crassirostris*	30	5:1	24.7	*Rhodomonas* sp. *T. weissflogii* *Heterocapsa* sp.	15,000 3,000 1,500	1.1

### Preparation of crude oil emulsions

Light Louisiana sweet crude oil was provided by BP (BP Exploration and Production, Inc.) as a surrogate for the Macondo (MC252) crude oil released in the DWH oil spill in the Gulf of Mexico (2010) because they are considered to have similar chemical composition and toxicity. The concentrations and composition of polycyclic aromatic hydrocarbons (PAHs) in this type of crude oil were previously determined by our research group and can be found in Almeda et al. (2013b). We used Corexit 9500A as the chemical dispersant because it was the main type of dispersant used in the clean-up operations during the DWH oil spill (National Commission on the BP Deep Ocean Horizon Oil Spill and Offshore Drilling 2011). The dispersant was provided by NALCO^®^ (Nalco/Exxon Energy Chemicals, L.P.) and some of its chemical ingredients can be found in the NALCO Environmental Solutions LLC web page (2010a).

We prepared three types of test media: (1) crude oil emulsions, i.e., suspensions of crude oil droplets in seawater dispersed physically without the addition of dispersant, (2) dispersant-treated crude oil emulsions i.e., crude oil emulsions in seawater dispersed physically and chemically and (3) a solution of dispersant alone in seawater. To prepare crude oil-seawater emulsions, 0.2 μm FSW was placed in a 1 L glass beaker with a magnetic stir bar, which was tightly sealed with aluminum foil to prevent oil absorption on the surface of the bar. The glass beaker containing the seawater was placed on a magnetic stirrer plate and stirred at 900 rpm. Then, 1 mL of crude oil was added to the seawater using an automatic pipette with a Pasteur glass pipette as a tip, that was thoroughly washed to remove the crude oil that could be attach to the pipette tip. After covering the beaker with aluminum foil, the crude oil was emulsified by keeping the stir rate at 900 rpm for 5 min at room temperature (25 °C). This stirring speed caused the formation of a vortex, which extends from the bottom of the container to the water surface, allowing the formation of crude oil droplets in seawater and keeping the crude oil emulsion homogenous during the mixing. The formation of oil droplets was confirmed in previous tests using an Imaging Particle Analysis system (FlowSight^®^). To prepare the dispersant treated-oil emulsions, we used the same methodology used for the preparation of the crude oil emulsions, but in this case we added 50 µL of chemical dispersant after adding the crude oil. We used a ratio of dispersant to oil of 1:20, which is in the range (1:50–1:10) recommended by the USEPA (1995). To prepare the dispersant solutions, 50 µL of chemical dispersant was added to 1 L of 0.2 μm FSW and stirred at 900 rpm for 5 min at 25 °C as in the preparation of the other test media. After the mixing time, 1 mL aliquots of each test medium were added to the corresponding 1 L experimental bottles to obtain the desired exposure concentration. The nominal concentrations used in the experiments were 1 µL L^−1^ for crude oil and dispersant-treated crude oil and 0.05 µL L^−1^ for dispersant.

### Experimental design and general procedures

Experiments consisted of 48 h laboratory incubations of single species of adult copepods exposed to crude oil alone (1 µL L^−1^), dispersant-treated crude oil (1 µL L^−1^) and dispersant alone (0.05 µL L^−1^) and in absence of pollutants (control treatments). We determined the acute effects of these pollutants on survival, EPRs, fecal pellet production rates (PPRs) and EH of the copepods, *A. tonsa*, *T. turbinata* and *P. crassirostris*. In the case of *A. tonsa*, we conducted two experiments, one in July and other in October. We used duplicates for each treatment in all the experiments except in the experiments conducted with *A. tonsa* in July when four replicates per treatment were run. The sex ratio and number of adult copepods used in each experiment are indicated in Table 1. Males and females were identificated according to their body morphology at the beginning of the incubation, except in the experiments conducted with *A. tonsa* in July, when the number of females and males added in each replicate/bottle was determined at the end of the incubation. Incubations were conducted in 1 L quartz bottles containing 0.2 µm-FSW (*S* = 34–35) and a mixture of cultured phytoplankton (Table 1). The cryptophyte *Rhodomonas* sp. (equivalent spherical diameter, ESD = 7.5 µm), the dinoflagellate *Heterocapsa* sp. (ESD = 16 µm), and the diatom *Thalassiosira weissflogii* (ESD = 14 µm) were the phytoplankton species used as food for the copepods (Table 1). Aliquots of cultured phytoplankton were added to the experimental bottles to obtain the food concentrations (in cell mL^−1^ and µg C mL^−1^) shown in Table 1. Phytoplankton cell volumes were calculated using the ESD and volumes were converted to carbon using the carbon to volume relationships established by Menden-Deuer and Lessard (2000). The concentration of phytoplankton in the cultures was determined with an inverted microscope (Olympus BX60) using a Sedgewick-Rafter counting chamber. After adding the crude oil emulsions/dispersant to the corresponding experimental bottles, all bottles were incubated on a Wheaton bench top roller (2–4 rpm) at 25 °C under dim light with a natural light–dark cycle.

After incubating, the contents of each bottle were gently screened through a submerged 150 µm mesh sieve to collect the copepods. Then, the sea water (<150 μm) containing copepod eggs, nauplii and fecal pellets was filtered using a 20 µm mesh sieve and placed in 20 mL glass containers. After the copepods were gently rinsed off the 150 µm mesh sieve, they were placed in glass dishes filled with 0.2 µm FSW for 5–10 min. We then checked the copepod survival and swimming activity by gently touching with a dissecting probe under a stereo microscope and determined the sex of the dead copepods in each replicate. Mortality, as % of the total incubated organisms, was estimated from the number of dead individuals at the end of the incubation (48 h). After determining the mortality of adult copepods, samples were fixed with glutaraldehyde (2 %) and kept at 4 °C, except for the experiments conducted in July with *A. tonsa* when samples were preserved in 1 % Lugol’s. To examine the presence of crude oil in the gut of the copepods, specimens were placed in glass chambers and observed under an epifluorescence microscope (Olympus BX51) with bright-field and UV illumination. The presence or absence of crude oil droplets in each copepod species was verified by the exposure to UV light (365 nm) that produces a strong fluorescence of crude oils due to their aromatic hydrocarbon fraction. Images of the copepods with both bright-field and UV illumination were captured with a digital camera attached to the microscope.

The number of eggs, copepod nauplii and fecal pellets in each sample were estimated under a stereomicroscope. The entire sample was counted to determine the number of nauplii and eggs. Egg production was estimated as the total number of eggs and hatched eggs (nauplii). Hatching (%) was assessed from number of nauplii in relation to total number of observed eggs and nauplii after incubation time. For the determination of the number of fecal pellets, an aliquot containing at least 100 fecal pellets (range 109–396) was fixed with Lugol’s (1 %) and counted under the stereomicroscope. EPR (eggs female^−1 ^day^−1^) and egestion rates (fecal pellets copepod^−1 ^day^−1^) were calculated considering only the number of live females and total live copepods, respectively, at the end of the incubation. The obtained data were expressed as average ± standard deviation and the significant differences among treatments were assessed using one-way analysis of variance (ANOVA) and least significant difference test (SPSS statistics 19.0 software).

## Results

**Fig. 1:**
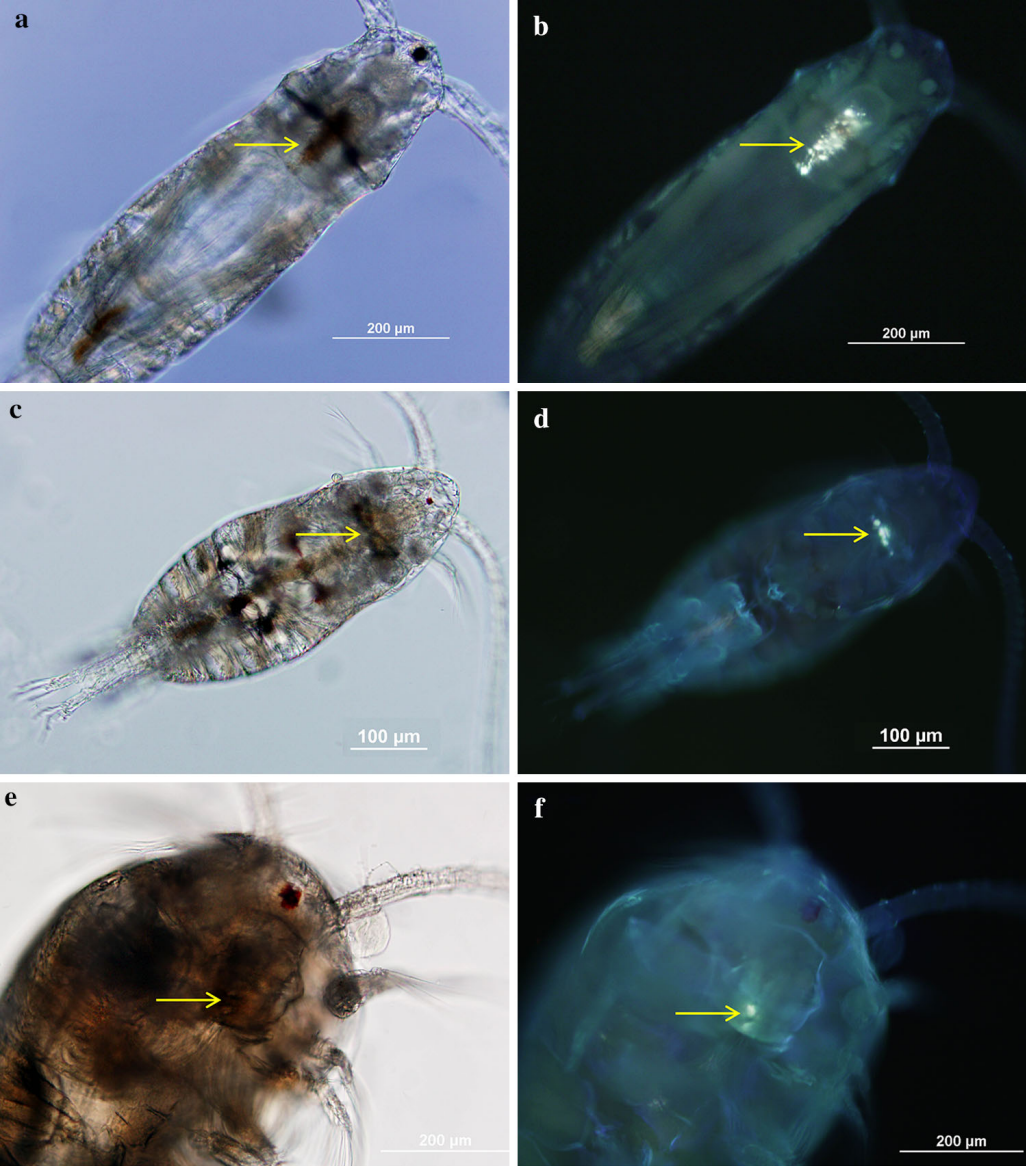
Microscope images of the studied copepods showing the presence of crude oil droplets inside the copepod digestive tracts after exposure to dispersed crude oil. The presence of crude oil droplets was confirmed by the observation of crude oil fluorescence under UV illumination (*right panels*). **a**, **b** *Acartia tonsa*, **c**, **d** *Parvocalanus crassirostris*, **e**, **f** *Temora turbinata*. The *arrow* indicates the position of crude oil droplets in the copepods

**Fig. 2:**
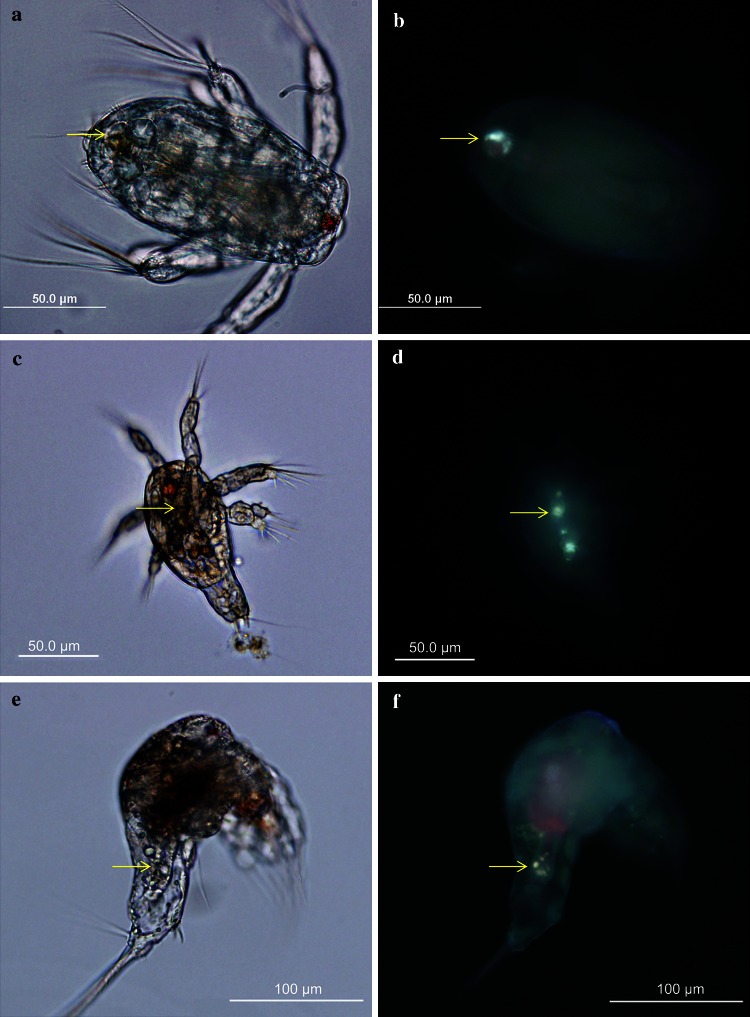
Microscope images of the copepod nauplii with crude oil droplets inside the guts. The presence of crude oil droplets was confirmed by the observation of crude oil fluorescence under UV illumination (*right panels*) **a**, **b** *Acartia tonsa*, **c**, **d** *Parvocalanus crassirostris*, **e**, **f** *Temora turbinata*. The *arrow* indicates the position of crude oil droplets in the copepods

Ingestion of crude oil droplets was observed in most specimens of three copepod species (Fig. 1) after exposure to both crude oil alone and dispersant-treated crude oil emulsions. We did not quantify the number of copepods with crude oil droplets inside their body but ~90–100 % of the copepod fecal pellets contained large amount of crude oil droplets, which indicate that all copepods were ingesting dispersed crude oil. Crude oil droplets were also observed inside the gut of some copepod nauplii (larval stage) of the three species (Fig. 2).

**Fig. 3:**
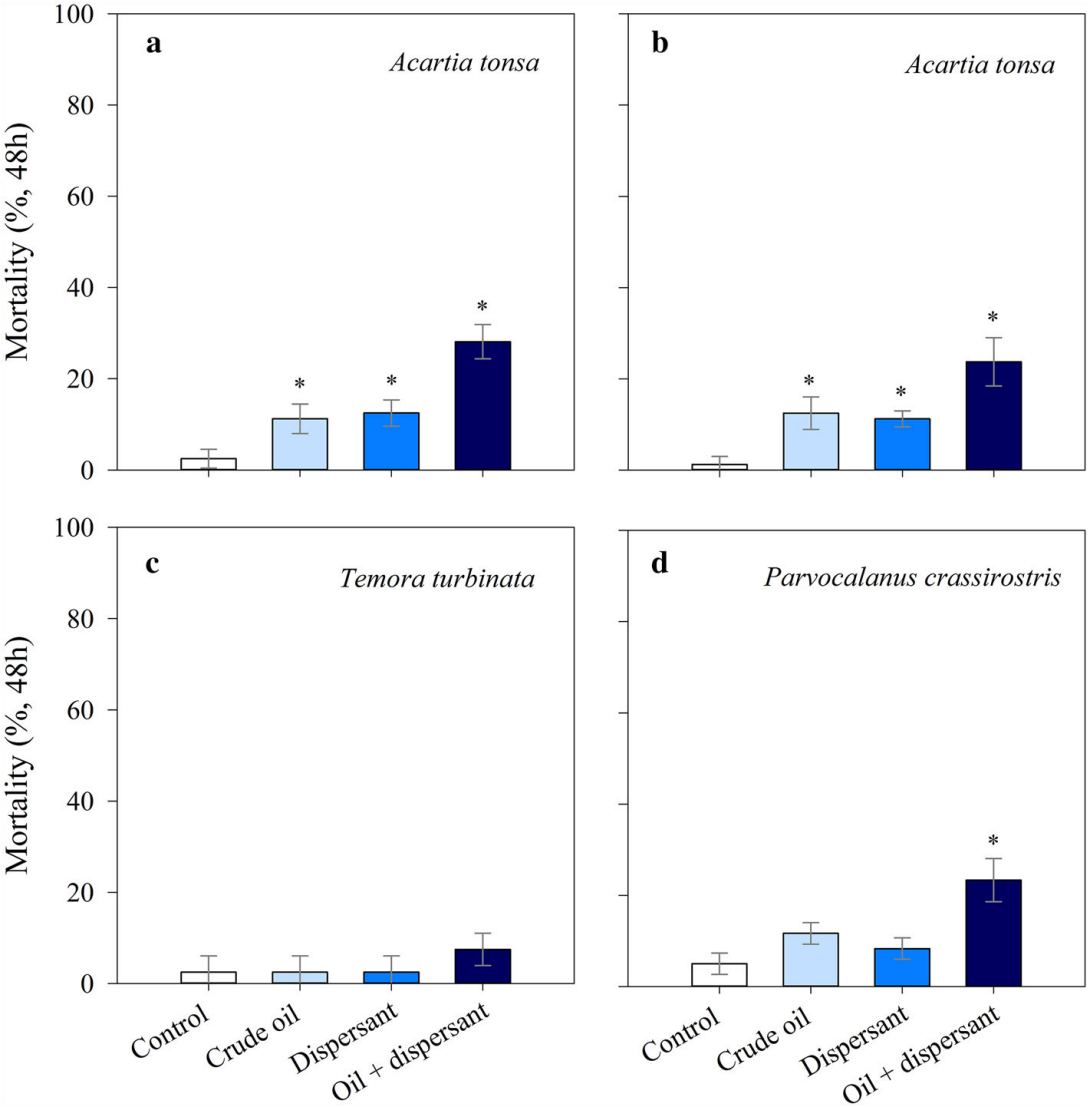
Lethal effects of crude oil alone, dispersant alone and dispersant-treated crude oil on copepods after 48 h of exposure. **a** *Acartia tonsa* (experiment conducted in July), **b** *Acartia tonsa* (experiment conducted in October), **c** *Temora turbinata*, and **d** *Parvocalanus crassirostris.* In all treatments *n* = 2, except in the experiments conducted with *A. tonsa* in July when *n* = 4. *Error bars* represent the standard deviations. *Asterisk* indicates significantly lower than the controls (*p* < 0.05)

Copepod average mortality in the experimental treatments ranged from 2.5 to 28 % depending on the species/experiments (Fig. 2). Survival of *A. tonsa* decreased significantly compared to controls after 48 h exposure to crude oil alone, dispersant and dispersant-treated crude oil in both experiments conducted with this species (July: Fig. 3a, ANOVA, *F*_3,12_ = 10.2, *p* < 0.01; October: Fig. 3b: ANOVA, *F*_3,4_ = 14.4, *p* < 0.05). In both experiments, mortality of *A. tonsa* after exposure to crude oil and dispersant alone, although higher than the controls, was low (~11–13 %, Fig. 3a, b), with no significant differences between these treatments (*p* > 0.05). Dispersant-treated crude oil caused the highest mortality of *A. tonsa* (~24–28 %), ~two times higher than the mortality caused by exposure to crude oil or dispersant alone (Fig. 3a, b). Survival of *T. turbinata* was unaffected or only slightly affected by 48 h exposure to crude oil, dispersant or dispersant-treated crude oil (Fig. 3c), with no significant differences among treatments including controls (ANOVA, *F*_3,4_ = 1, *p* = 0.48). Similar to *A. tonsa*, mortality of *P. crassirostris* was significantly higher when exposed to dispersant-treated crude oil than in the other treatments (ANOVA, *F*_3,4_ = 13.1, *p* < 0.05), ca. two times higher than when copepods were exposed to crude oil alone (Fig. 3d). Average mortality of *P. crassirostris* in the crude oil and dispersant alone treatment was higher, but not significantly different, than in the control (Fig. 3d, ANOVA *p* > 0.05).

**Fig. 4:**
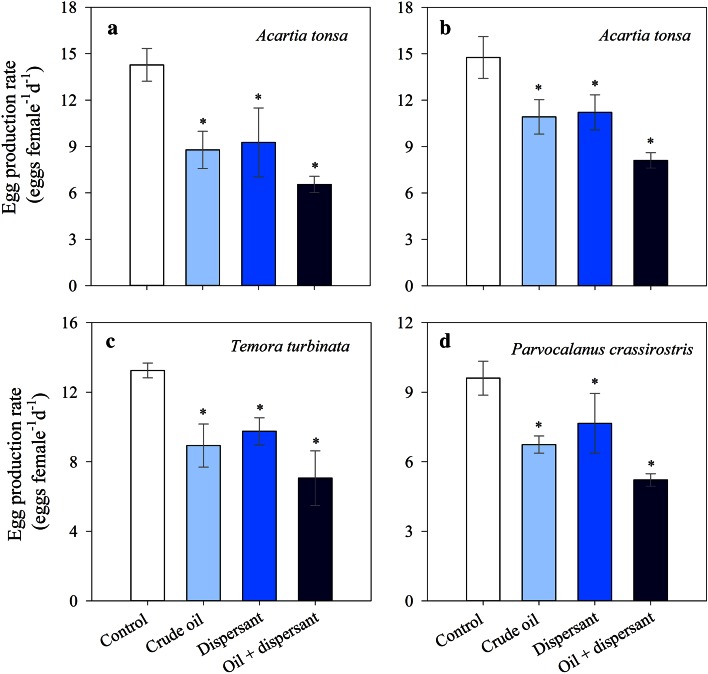
Effects of crude oil alone, dispersant alone and dispersant-treated crude oil on copepod egg production rates after 48 h of exposure. **a** *Acartia tonsa* (experiment conducted in July), **b** *Acartia tonsa* (experiment conducted in October), **c** *Temora turbinata*, and **d** *Parvocalanus crassirostris.* In all treatments *n* = 2, except in the experiments conducted with *A. tonsa* in July when *n* = 4. *Error bars* represent the standard deviations. *Asterisk* indicates significantly lower than the controls (*p* < 0.05)

EPRs (eggs female^−1 ^day^−1^) varied from ~5 to 15 depending on the species and treatments (Fig. 4). We observed that exposure to crude oil alone, dispersant or dispersant-treated crude oil caused a reduction in EPR of the three copepod species compared to their respective controls (Fig. 4). EPR of *A. tonsa* were significantly lower in all experimental treatments than in the controls (July: Fig. 4a, ANOVA, *F*_3,11_ = 18.6, *p* < 0.01; October: Fig. 4b, ANOVA, *F*_3,4_ = 12.9, *p* < 0.05). Exposure to crude oil caused a reduction in *A. tonsa* EPR by 39 and 26 % in the July and October experiments, respectively, compared with the controls (Fig. 4a; Table 2). EPR of *A. tonsa* were reduced by 35 and 24 % after exposure to dispersant alone (Fig. 4b). However, there was no significant difference in EPR between these two experimental treatments for either July and October experiments (Fig. 4a, b; *p* > 0.05). Exposure to dispersant-treated crude oil caused the highest reduction in *A. tonsa* EPR, by 54 % in July (Fig. 4a) and 45 % in October (Fig. 4b). This reduction in EPR was 1.4 and 1.7 times higher compared to crude oil alone for July and October experiments, respectively (Fig. 4a, b). EPR of *T. turbinata* were significantly reduced by 33, 26 and 47 % after exposure to crude oil, dispersant and dispersant-treated crude oil, respectively, compared to the control (Fig. 4c; ANOVA, *F*_3,4_ = 11.2, *p* < 0.05). Although lower than controls, no significant differences in *T. turbinata* EPR were observed among all three experimental treatments (Fig. 4c, *p* > 0.05). EPR of *P. crassirostris* were significantly lower in the experimental treatments than in the control (Fig. 4d, ANOVA, *F*_3,4_ = 11.1, *p* < 0.05), except for the dispersant treatment, where no significant differences were observed (Fig. 4d; *p* > 0.05). Exposure to crude oil and dispersant alone caused a reduction in *P. crassirostris* EPR by 30 and 20 %, respectively (Fig. 4d), with no significant differences between these two experimental treatments (Fig. 4d; *p* > 0.05). As observed in the other copepod species, exposure to dispersant-treated crude oil caused the highest reduction in EPR of *P. crassirostris*, by 46 % compared to the control, and 1.5 times higher than the crude oil alone treatment (Fig. 4d).

**Table 2:** Percent (%) of reduction in copepod egg production rates (EPR), fecal pellet production rates (PPR) and egg hatching (EH) in the experimental treatments compared to the controls

Species/experiments	EPR			PPR			EH		
	Crude oil	Disp.	Oil + disp.	Crude oil	Disp.	Oil + disp.	Crude oil	Disp.	Oil + disp.
*A. tonsa_*July	39*	35*	54*	17*	23*	38*	17*	11	22*
*A. tonsa_*Oct	26*	24*	45*	23*	19*	41*	6	7*	15*
*T. turbinata*	33*	26*	47*	11*	15*	28*	1	5	11*
*P. crassirostris*	30*	20	46*	27*	12	37*	38*	2	31*

Asterisk indicates significantly lower than the controls (*p* < 0.05)

**Fig. 5:**
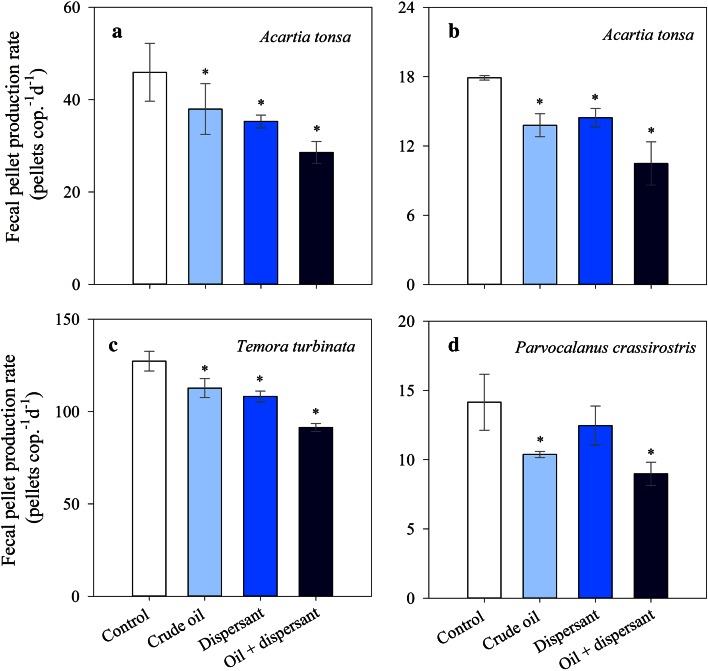
Effects of crude oil alone, dispersant alone and dispersant-treated crude oil on copepod fecal pellet production rates after 48 h of exposure. **a** *Acartia tonsa* (experiment conducted in July), **b** *Acartia tonsa* (experiment conducted in October), **c** *Temora turbinata*, and **d** *Parvocalanus crassirostris*. In all treatments *n* = 2, except in the experiments conducted with *A. tonsa* in July when *n* = 4. *Error bars* represent the standard deviations. *Asterisk* indicates significantly lower than the controls (*p* < 0.05)

Fecal PPRs (pellets cop^−1 ^day^−1^) ranged from ~8 to 127 depending on the species and treatments (Fig. 5). PPRs of copepods were significantly lower in all experimental treatments than in the controls (Fig. 5, ANOVA, *p* < 0.05), except for *P. crassirostris*, where no significant differences were observed between the dispersant alone treatment and the control (Fig. 5d; *p* > 0.5). In the experiment conducted with *A. tonsa* in July, PPR were 17 and 23 % lower after exposure to crude oil and dispersant, respectively, compared with the control (Fig. 5a), and 23 and 10 % lower in the experiment conducted in October (Fig. 5b). PPR of *T. turbinata* after exposure to crude oil and dispersant were 11 and 15 % lower, respectively, than in the controls (Fig. 5c; ANOVA, *F*_3,4_ = 25.9, *p* < 0.01). Exposure to dispersant-treated crude oil caused the highest reduction in PPR of *T. turbinata*, by 28 % compared to the controls and 1.5 times higher than in the crude oil alone treatment. Exposure to crude oil and dispersant alone caused a reduction in *P. crassirostris* PPR by 27 and 12 %, respectively (Fig. 5d). For all four experiments with the three species, no significant differences in copepod PPR were found between the crude oil and dispersant treatments (Fig. 5; ANOVA *p* > 0.5). Exposure to dispersant-treated crude oil caused the highest reduction in PPR for all three species of copepods, 38 % for *A. tonsa* in July (Fig. 5a), 41 % for *A. tonsa* in October (Fig. 5b), 28 % for *T. turbinata* (Fig. 5c), and 37 % for *P. crassirostris* (Fig. 5d), which represent PPR between 1.4 and 2.5 times lower than in their corresponding crude oil alone treatments (Fig. 5). In the three species the reduction on PPRs was lower than the reduction in EPRs.

**Fig. 6:**
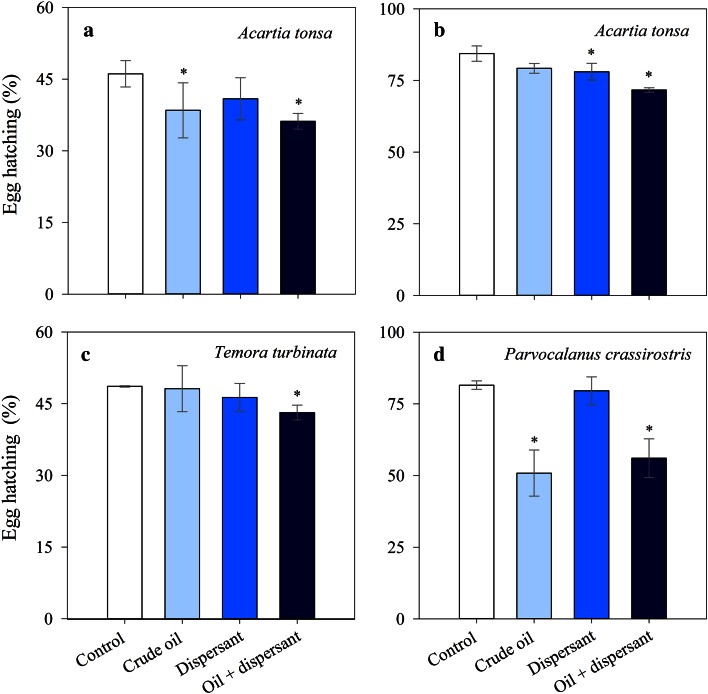
Effects of crude oil alone, dispersant alone and dispersant-treated crude oil on copepod egg hatching after 48 h of exposure. **a** *Acartia tonsa* (experiment conducted in July), **b** *Acartia tonsa* (experiment conducted in October), **c** *Temora turbinata*, and **d** *Parvocalanus crassirostris*. In all treatments *n* = 2, except in the experiments conducted with *A. tonsa* in July when *n* = 4. *Error bars* represent the standard deviations. *Asterisk* indicates significantly lower than the controls (*p* < 0.05)

Copepod EH success ranged from 33 to 87 % depending on the species/experiments and treatments. The effect of the crude oil alone and dispersant alone on EH was very variable and depended on species (Fig. 6; Table 2). In the case of *A. tonsa*, exposure to crude oil and dispersant caused a reduction in EH by 6–17 % compared to the control (Fig. 6a, b; Table 2) with no significant differences among experimental treatments (*p* > 0.05). EH of *T. turbinata* was unaffected or only slightly affected by exposure to crude oil or dispersant, but EH was significantly lower (11 %) after exposure to dispersant-treated crude oil compared to the control (Fig. 6c, *F*_1,2_ = 24.6, *p* < 0.05; Table 2). We did not find significant effects on EH of *P. crassirostris* after exposure to dispersant alone (Fig. 6d). However the exposure to crude oil alone and dispersant-treated crude oil caused a significant reduction in EH by 38 and 31 %, respectively, in *P. crassirostris* compared to the control (Fig. 6d, ANOVA, *p* < 0.05; Table 2). No significant difference in EH of *P. crassirostris* were observed between crude oil alone and dispersant-treated crude oil treatments (Fig. 6d, ANOVA, *p* > 0.05).

Comparing among species, *T. turbinata* showed a higher survival and lower reduction in fecal PPRs and EH after exposure to crude oil, dispersant or dispersant-treated crude oil than the other copepods (Fig. 3; Table 2). However, the reduction in egg production in the experimental treatments was quite similar among species (Table 2).

## Discussion

Our results indicate that acute exposure to low, sublethal concentrations of dispersed crude oil and Corexit 9500A produce a substantial reduction in the reproduction and egestion rates of important species of marine planktonic copepods. The three species of copepods studied here are ecologically relevant for the Gulf of Mexico. Therefore, our results are particularly valuable for understanding the potential impact of crude oil pollution on plankton communities from the Gulf of Mexico, a region with a high risk for crude oil spills due to the intense petroleum industry activities in these waters. Our results support previous studies than indicate that zooplankton are particularly vulnerable to the impact of catastrophic crude oil spills (Moore and Dwyer 1974; Lee 1977; Johansson et al. 1980; Avila et al. 2010; Almeda et al. 2013a, b). Given the key role of copepods in marine food webs and their high sensitivity to crude oil and dispersant, planktonic copepods should be used as target group to evaluate the toxicity and environmental impact of crude oil spills on marine environments.

Crude oil exposure concentration is one of the main factors affecting the toxicity of crude oil to marine organisms. Generally, toxicity of crude oil increases as oil concentration increases. After catastrophic crude oil spills, concentrations of crude oil in the water column are highly variable, both spatially and temporarily, making accurate measurements of crude oil concentration in the sea difficult. Depending on the marine topography and hydrodynamics (e.g., mixing energy caused by wind and currents) and if chemical dispersants are applied to treat the crude oil spill, planktonic organisms can be exposed to crude oil concentrations ranging from more than 200 ppm to less than 1 ppb (Lichtenthaler and Daling 1985; McAuliffe et al. 1981; Clayton et al. 1993; Kerr 2010; Whitehead et al. 2011). The exposure levels of crude oil used in this study (1 µL L^−1^, ~0.85 ppm) are in the range of concentrations commonly found in the water column after oil spills. For example, plumes of dispersed crude oil at concentrations of 1–2 ppm were observed at 1 km depth after the DWH crude oil spill in the Gulf of Mexico (Kerr 2010). The exposure concentration of crude oil used in this study (1 µL L^−1^) corresponds to a total PAHs concentration of ~2.15 ppb, based the concentration of PAHs previously determined in this oil (2.15 µg µL^−1^; Almeda et al. 2013b). This PAH concentration is in the lower range for concentrations commonly found in the water column after oil spills (from less than 1 ppb to more than 150 ppb), including the DWH crude oil spill (Barbier et al. 1973; Neff and Stubblefield 1995; Short and Rounds 1993; Law et al. 1997; Wade et al. 2011). Similarly, although direct field measurements of dispersant concentrations during oil spills are scarce, the concentration of dispersant used in this study (0.05 µL L^−1^, equivalent to ~48.4 ppb) is in the lower range of dispersant concentrations estimated after field applications (from less than 1 ppm to more 10 ppm; Bocard et al. 1984; Mackay and Hossain 1982; Wells 1984) and in the same order of magnitude of the dispersant concentrations used during the DWH oil spill according to estimations provided by NALCO Environmental Solutions LLC (2010b; ~30 ppb). It is important to note that, in the natural environment, toxicity of crude oil not only depends on the concentration of crude oil and duration of exposure but also on environmental conditions. Consequently, the impact of catastrophic crude oil spills on plankton will vary depending on the specific circumstances of each accident. For instance, temperature and UV radiation may increase substantially the toxicity of crude oil to marine zooplankton (Duesterloh et al. 2002; Jiang et al. 2012; Almeda et al. 2013a). Therefore, the impact of crude oil on zooplankton may be higher in warm seasons/areas with elevated UV radiation. Even though the extrapolation of specific laboratory studies to the field needs to be taken cautiously, toxicological laboratory/experimental studies are a reliable means of detecting important toxic effects of petroleum on zooplankton. Therefore, our results further our understanding of the acute effects of physically and chemically dispersed crude oil on marine copepods under realistic crude oil and chemical dispersant concentrations after catastrophic oil spills, helping to predict the potential impacts of crude oil pollution on the marine plankton.

Previous laboratory studies have also found that acute exposure to petroleum hydrocarbons caused lethal and sublethal effects on zooplankton in agreement with our results (Lee 1977; Gyllenberg and Lundqvist 1976; Bellas and Thor 2007; Avila et al. 2010; Almeda et al. 2013a). Lethal and sublethal effects on copepods may vary depending on the copepod species, methodology and experimental conditions. For example, a recent study (2012) on the lethal effects of crude oil WSF on copepods found that body size was inversely correlated with crude oil toxicity (Jiang et al. 2012). This size relationship would be one reason, along with interspecies genetic variability, that explains the differences in toxicity observed among the three copepods studied here, where the small–medium sized copepods *A. tonsa* and *P. crassirostris* were more affected by dispersed crude oil exposure than the larger copepod *T. turbinata.* The observed differences in fecal PPRs, egg production and EH among the different controls for the different experiments may not only be due to interspecific variation, but also to the difference in experimental food conditions, which may affect the egg production, egestion rates and EH success of copepods (Kleppel and Burkart 1995; Feinberg and Dam 1998). Therefore, it is important to note that estimations of the effects of crude oil exposure on the studied vital rates should only be done using the corresponding control that had the same specific experimental conditions. Differences in EPRs and egestion rates observed among the different treatments in each experiment are not related to effects of oil on prey abundance, since the phytoplankton used in these experiments have a higher tolerance to crude oil than zooplankton, according to our observation and previous studies (Prouse et al. 1976; Morales-Loo and Goutx 1990; Echeveste et al. 2010; Jiang et al. 2010). The reduction in egestion rates observed for these copepods may be related to a decrease in ingestion rates due to narcosis and behavior effects of crude oil exposure on copepods (Gyllenberg and Lundqvist 1976; Berdugo et al. 1977; Berman and Heinle 1980; Cowles and Remillard 1983; Avila et al. 2010). The ingestion of crude oil droplets, as reflected in faecal pellets containing large amounts of crude oil droplets, may also affect the gut transit or egestion rates of copepods. Among the different vital processes studied, EPRs seem to be more affected than egestion rates and EH. Detrimental effects of crude oil on behavior, energetics and biochemical processes associated with reproduction may explain the reduced egg production observed in planktonic copepods (Saiz et al. 2009; Avila et al. 2010; Seuront 2011). For example, narcosis and behavior effects could cause a reduction in feeding efficiency, thereby reducing the amount of resources that can be allocated for producing eggs. Similarly, the ingestion of crude oil droplets may affect the assimilation efficiency of nutrients and consequently negatively impact egg production. Also, exposure to petroleum hydrocarbons may decrease mating success in planktonic copepods (Seuront 2011), which is also likely to affect EPRs. Alteration in lipid metabolism after exposure to petroleum hydrocarbons, such as in steroid metabolism, may produce anomalies in reproduction and development in crustaceans (Singer and Lee 1977), likely contributing to reduction in hatching success of copepods when exposed to crude oil. The negative impact of crude oil on copepod reproductive success (both egg production and EH) has immediate consequences for nauplii recruitment. This outcome is particularly important for fish production, since copepod nauplii are the main food of many fish larvae and their abundance determines the recruitment of commercially important fish species (Last 1980; Castonguay et al. 2008). However, since fish production not only depends on zooplankton abundance, additional factors have to be considered to more fully understand the impact of oil spills on higher trophic levels. Overall, our results indicate that dispersed crude oil caused acute significant sublethal effects on key species of planktonic copepods, which may affect zooplankton population dynamics and consequently secondary production in marine environments.

Several field studies have reported short- and long-term decreases in zooplankton concentrations after oils spills (Johansson et al. 1980; Samain et al. 1980; Guzmán del Próo et al. 1986), which supports our conclusion that crude oil pollution may negatively affect zooplankton population dynamics. Although negative short-term effects of crude oil spills on zooplankton are generally acknowledged, long-term effects of crude oil pollution and the capacity for recovery by zooplankton communities are still important questions needing further attention (Olsen et al. 2013). The long-term impact of oil on zooplankton communities likely depends on the specific environmental characteristics of the affected area and species composition of the planktonic community. For example, some species of pelagic copepods release their eggs at distinct times of year. This seasonal egg release may include the production of resting eggs during the phytoplankton growing season, eggs that remain in the sediments until the following year (Marcus 1996). Similarly, spawning of marine benthic invertebrates shows strong seasonality, with distinct seasonal peaks of egg and planktonic larvae abundance (Thorson 1950; Highfield et al. 2010). If an oil spill affects these organisms during their spawning season, reduced egg production and larval survival will adversely influence recruitment for the following year, and therefore negatively impacting population dynamics of planktonic and benthic communities. These examples underscore the complexity of evaluating long-term effects of oil spills on zooplankton communities, and their ecological impact in marine environments. Additional field and laboratory studies are required to increase our understanding of the long-term effects of crude oil on planktonic communities.

Since the application of large amounts of Corexit dispersants in the DWH crude oil spill, there have been increasing interest and discussion about the effects of these dispersants to marine life (USEPA 2010; Wise and wise 2011; NALCO^®^ Environmental Solutions 2010b). Toxicity of chemical dispersants is associated with their chemical components, such as solvents, surfactants and additives. The toxic mechanisms of dispersants are not fully understood but surfactants can affect cellular membranes, increasing membrane permeability and causing membrane lysis in marine organisms (Nagel et al. 1974; Singer et al. 1990). After the Torrey Canyon (1967) and Sea Empress (1966) crude oil spills, where the application of old types of dispersants caused dramatic environmental damage (Corner et al. 1968; Nelson-Smith 1968; Swedmark et al. 1973), new formulations of chemical dispersants, such as Corexit 9500A, had been developed. Based on certain toxicological studies, it has been suggested that the new generation of dispersants and dispersant-treated crude oil are less toxic than crude oil alone (George-Ares and Clark 2000; Lewis 2001; Hemmer et al. 2010) and that they have minimal deleterious effects on marine life (Lessard and Demarco 2000). However, there is a gap in our knowledge of the effects of chemical dispersants on marine zooplankton, particularly on copepods. Even though Corexit 9500A is less toxic than previous dispersant types to certain marine organisms (Singer et al. 1996), our results demonstrate that this type of dispersant is toxic to planktonic copepods even at low exposure concentration (~48 ppb), causing lethal and sublethal effects slightly lower than crude oil alone. In addition, in a recent study, 48 h exposure to Corexit 9500A at 0.25 ppm caused nearly 50 % mortality of mesozooplankton, which were mainly dominated by planktonic copepods (Almeda et al. 2013a). This lethal concentration is more than one order of magnitude lower than lethal concentrations commonly observed in other marine animals exposed to chemical dispersant (Singer et al. 1995, 1996). In agreement with our results, recent laboratory studies found that at low concentrations Corexit 9500A is also toxic to other small planktonic organisms, fish eggs and larvae (Barron et al. 2003), coral larvae (Goodbody-Gringley et al. 2013), and rotifers (Rico-Martinez et al. 2013). Further, our work demonstrates that this type of dispersant is more toxic to zooplankton than previously assumed.

After a crude oil spill, the application of chemical dispersants enhances the formation of small stable crude oil droplets (Lichtenthaler and Daling 1985; Delvigne and Sweeney 1988; Mukherjee and Wrenn 2009), increasing the potential for planktonic organisms to interact with dispersed crude oil. One of the chief conclusions of this study is that chemically dispersed crude oil is more toxic than physically dispersed crude oil to planktonic copepods. Studies of the effects of dispersant-treated crude oil on zooplankton are very scarce and sometimes controversial (Linden et al. 1987; Jung et al. 2012). However, there is increasing evidence that the combination of oil and dispersant increases toxicity of crude oil to marine organisms, such as fish larvae and eggs, and other planktonic organism, in agreement with our results (Barron et al. 2003; Jung et al. 2012; Goodbody-Gringley et al. 2013; Rico-Martinez et al. 2013). Increased toxicity of dispersant-treated crude oil is associated with both additive and/or synergistic effects of oil and dispersant. As demonstrated here, Corexit 9500A dispersant is itself toxic to marine copepods. Further toxicity from the application of a chemical dispersant after an oil spill can result from an increase in the dissolution of toxic soluble components of crude oil, like PAHs in the water (Greer et al. 2012; Wu et al. 2012). However, in our experiments, the observed reduction on survival and physiological rates of copepods after exposure to dispersant-treated crude oil seems to be mainly due to the additive toxicity of crude oil and dispersant (Table 2). In some experiments, the sum of reduction on vital rates from crude oil and dispersant alone treatments is slightly higher than the reduction in the dispersed crude oil treatment. One possible explanation is that EPRs and fecal PPRs in this study were calculated using the number of live specimens at the end of the incubation. Since these specimens were probably producing eggs and fecal pellets before dying, especially within the first several hours of the incubation, the sublethal effects may be underestimated, particularly in the dispersed treated crude oil treatment when mortality was higher (Table 2). Overall, our results indicate that copepods are negatively affected by dispersant Corexit 9500A and chemically dispersed crude oil. This emphasize the need for more studies on the effects on dispersant and dispersed crude oil on key zooplankton groups that are currently understudied (e.g. copepod nauplii, meroplankton, ciliates, etc.) to better understand the impact of dispersants and dispersed crude oil on planktonic communities.

After a crude oil spill, petroleum is present in the water column in both dissolved and particulate (i.e. crude oil droplets) forms. As mentioned in the “Introduction” section, most crude oil toxicological research has been conducted with the WSF or individual or mixed dissolved petroleum hydrocarbons (Berdugo et al. 1977; Barata et al. 2005; Bejarano et al. 2006; Calbet et al. 2007; Saiz et al. 2009; Jiang et al. 2010, 2012). However, our results confirm that copepods take up petroleum hydrocarbons not only through passive mechanisms from dissolved petroleum hydrocarbons or contaminated phytoplankton, but also through the ingestion of crude oil droplets as observed in this study. Conover (1971) was one of the first to notice that some copepods ingested crude oil droplets after an accidental crude oil spill. Since then, there has been increasing evidence of the ingestion of particulate crude oil by copepods and other zooplankton according to laboratory studies and field observations (Andrews and Floodgate 1974; Mackie et al. 1978; Hebert and Poulet 1980; Gyllenburg 1981; Lee et al. 2012). As far we know, this is the first report of ingestion of crude oil droplets by these important species of planktonic copepods and copepod nauplii. It is important to note that ingestion of crude oil droplets has been frequently associated with feeding-current feeder zooplankton (Lee et al. 2012), and not to ambush feeders such as *Acartia tonsa* nauplii. The high number of adult copepod fecal pellets containing crude oil droplets indicates that all the adult copepods ingested dispersed crude oil. We did not examine fecal pellets of copepod nauplii and further research is required to quantify the ingestion of crude oil droplets by copepod nauplii. The presence of crude oil in copepods or fecal pellets may be difficult to observe with bright light under the microscope given that the color and morphological characteristics of crude oil droplets are similar to other lipids and components, or because gut contents and fecal pellets are densely packed. The use of fluorescence of crude oil under UV illumination, as used in this study, is a useful tool to help determine the presence of crude oil droplets in zooplankton guts or fecal pellets.

We found that dispersed crude oil caused greater lethal and sublethal effects on copepods at equal or lower concentrations than those of dissolved petroleum hydrocarbons (Berdugo et al. 1977; Barata et al. 2005; Bejarano et al. 2006; Calbet et al. 2007; Saiz et al. 2009; Jiang et al. 2010, 2012). This suggests that ingestion of crude oil droplets may increase the toxicity of petroleum to marine copepods. As compared to experiments using WSF or single PAHs, exposure to dispersed crude oil may enhance zooplankton uptake of PAHs, particularly those hydrocarbons with low solubility (Ramachandran et al. 2004), which are frequently more toxic than more soluble and volatile PAHs (e.g. naphthalene; Berdugo et al. 1977; Barata et al. 2002, 2005). The presence of crude oil droplets in the fecal pellets observed in our experiments supports previous studies that found accumulation of petroleum hydrocarbons in zooplankton faecal pellets (Prahl and Carpenter 1979; Sleeter and Butler 1982; Almeda et al. 2013a). The use of dispersed crude oil represents a more realistic scenario than the use WSF to study the interactions between crude oil and zooplankton after oil spills. Future research on the quantification of the amount of dispersed crude oil ingested, accumulated and defecated by zooplankton is necessary.

Our results support the notion that ingestion of crude oil droplets by zooplankton is not an anecdotic event and should not be ignored in crude oil pollution studies. At low, sublethal concentrations of dispersed crude oil, as observed frequently in large plumes after crude oil spills, we expect that copepod are able to survive, ingest crude oil droplets and produce crude oil contaminated fecal pellets. The ingestion of dispersed crude oil by copepods may increase uptake, biotransfer and biomagnification of highly toxic, low soluble PAH through food webs. Therefore, more research on the quantification of the amount of dispersed crude oil ingested by zooplankton and their consequences for the toxicity and bio-transfer of petroleum through food webs is required to better understand the impact and fate of crude oil pollution on marine environments.
